# This Is Not a Christmas Editorial!

**DOI:** 10.32872/cpe.v2i4.5433

**Published:** 2020-12-23

**Authors:** Cornelia Weise, Winfried Rief

**Affiliations:** a Division of Clinical Psychology and Psychotherapy, Department of Psychology, Philipps-University of Marburg, Marburg, Germany

When the two editors-in-chief of this journal met to discuss whether we should strive for a Christmas editorial this year, it was a moment of desperation. We began brainstorming potential topics over Zoom. It had been the fourth video conference on a Friday afternoon for both of us, and we were starting to experience headaches, fatigue, vision and concentration problems, as well as annoying noises in the ear. And we also felt that we are tired of speaking and writing about all the events that characterized this very special year. Last but not least we considered: Is a Christmas editorial still contemporary and fresh, especially if we want to express our openness to the diversity of people, cultures, and religions?

We started by investigating the background of Christmas, and we did it empirically (what else would you expect?). A word search in the Bible with typical, Christmas-associated items seemed a good way to start to evaluate religious chauvinism. However, neither “Rudolph, the red nosed reindeer” nor “Christmas tree” led to any hits. Wikipedia informed us that the Christmas tree goes back to the days of Nordic tribes, and many rituals of the end-of-the-year season have their roots in profane rites of celebrating the longest night. After a period of searching, even the origin of Santa Claus became more and more blurred. The white-bearded male with a BMI of >35 does not resemble any head of most popular religions in Europe. Eventually we decided that writing a Christmas editorial is not cultural chauvinism, but rather that it is more of a chance to reflect before the year ends on what has happened in 2020. But to be on the safe side: This is not a Christmas editorial.

So what should we write about? Many themes came to mind that we were tired to talk or write about. A virus posed a threat to all of us, but are our readers really keen to hear even more about the C-word at the very end of the year? Although some aspects are still worth mentioning, even in a Not-a-Christmas-Editorial. This year we learned so much about Zooming, break-out rooms, and personal background preferences. What, for example, is the clinical implication of people preferring a Spiderman-virtual-background vs. a fake office background, or how relevant are the number of kids and cats appearing in the speaker's background? Is it really true that nations differed in terms of the choice of goods hoarded during lockdowns? Are these preferences really related to the people's mental health status in the respective countries, e.g. weed in the Netherlands, feta cheese in Greece, toilet paper in Germany, or wine and condoms in France? With a clear preference to migrate to France, we ended this discussion.

Now is the time to stand together and solve problems. Stop, no. We have to respect physical distancing. But why is there a country in Europe who really exaggerates social distancing? Shall we dedicate a special paper to this topic in our “Politics and education” section? How do we maintain the illusion of independent countries in the 21st century? But no, as with the C-word, we do not want to talk about the B-word either; we express our sympathies to all European and Non-European countries, even if they are on islands drifting around in the North Sea. You are always very welcome to join us under the umbrella of our journal.

Finally, in our discussion we turned to the US elections. Although we are not a political journal, this topic offers many possible starting points for an Editorial. For example, it would have provided perfect examples for psychological treatment (e.g. behaviour analysis, reality neglect, cognitive reframing and working with infantile schema modes). Since classification of mental disorders started more than 100 years ago, the starting point of classifying mental disorders was always the neglect of reality: medium in neuroses, even more serious in psychosis. However, while ruminating about these topics, we became more and more worried that we would end up writing a comprehensive overview of personality disorders, which is too big a topic, and beyond our expertise.

Therefore, we decided to write just a brief editorial with two major points. First, we want to express our thanks to all people involved in CPE's second volume. We wish to acknowledge our authors, reviewers, section editors, guest editors, and the whole publishing team. When we started with this journal more than two years ago, we were worried how the new publication would be accepted by the scientific community? It is risky to start a new scientific journal, in an era when scientists receive daily announcements and requests to submit to obscure and unknown journals.

But we did it. And it has been a really successful year for CPE. Not only have we published the first two volumes, but there are also a number of exciting manuscripts in the pipeline, and further manuscripts are waiting for consideration. We are grateful for all the support we have received – particularly in this tough and challenging year – and we are extremely pleased to see CPE becoming a more and more impactful journal.

Second, we want to wish you a Happy Holiday season and a peaceful and prosperous New Year. We hope it will soon be possible to meet up with family and friends, to pursue hobbies, and to do all the things that belong to our previous regular life. We all know about the challenges of the current times, and should therefore use our knowledge and expertise in clinical psychology to get through this challenging time together and support those needing help.

We are looking forward to receiving your submissions in 2021 despite all the challenges that we are all facing. Thank you for your support of the journal.

**Figure:**
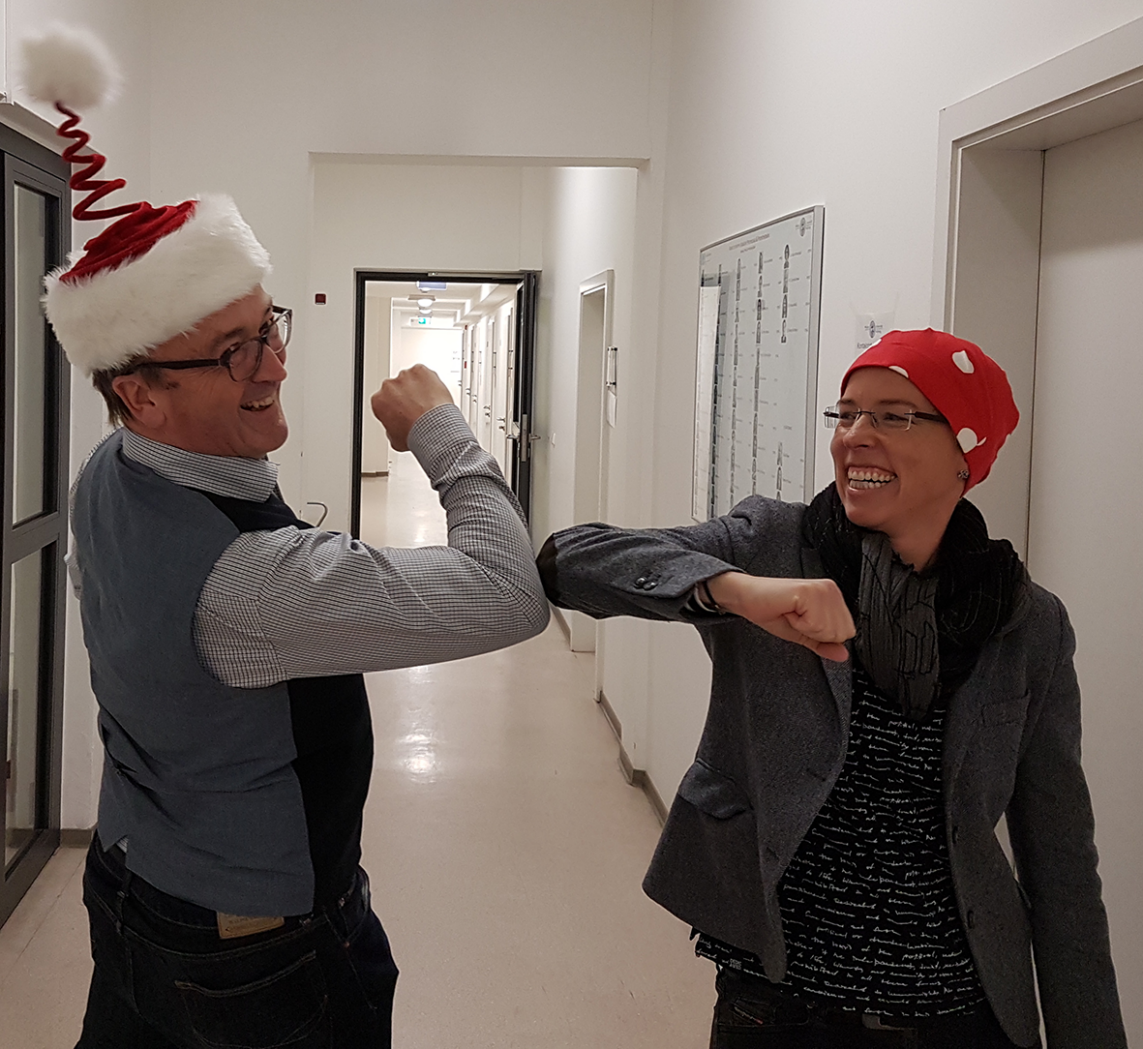
Pandemic-Compliant Greetings to All of You



**Cornelia Weise & Winfried Rief**



